# Functional connectivity of the cerebellar vermis in bipolar disorder and associations with mood

**DOI:** 10.3389/fpsyt.2023.1147540

**Published:** 2023-05-05

**Authors:** Arshaq Saleem, Gail Harmata, Shivangi Jain, Michelle W. Voss, Jess G. Fiedorowicz, Aislinn J. Williams, Joseph J. Shaffer, Jenny Gringer Richards, Ercole John Barsotti, Leela Sathyaputri, Samantha L. Schmitz, Gary E. Christensen, Jeffrey D. Long, Jia Xu, John A. Wemmie, Vincent A. Magnotta

**Affiliations:** ^1^Department of Psychological and Brain Sciences, University of Iowa, Iowa City, IA, United States; ^2^Department of Radiology, University of Iowa, Iowa City, IA, United States; ^3^The Ottawa Hospital, Ottawa Hospital Research Institute, University of Ottawa Brain and Mind Research Institute, Ottawa, ON, Canada; ^4^Department of Psychiatry, University of Iowa, Iowa City, IA, United States; ^5^Department of Biosciences, Kansas City University, Kansas City, MO, United States; ^6^Embracing the World, Elburn, IL, United States; ^7^College of Osteopathic Medicine, Des Moines University, Des Moines, IA, United States; ^8^Department of Electrical and Computer Engineering, University of Iowa, Iowa City, IA, United States; ^9^Department of Radiation Oncology, University of Iowa, Iowa City, IA, United States; ^10^Department of Biostatistics, University of Iowa, Iowa City, IA, United States; ^11^Veterans Affairs Medical Center, Iowa City, IA, United States; ^12^Department of Molecular Physiology and Biophysics, University of Iowa, Iowa City, IA, United States; ^13^Department of Neurosurgery, University of Iowa, Iowa City, IA, United States; ^14^Department of Biomedical Engineering, University of Iowa, Iowa City, IA, United States

**Keywords:** bipolar disorder, cerebellar vermis, resting state functional imaging, mood disorders, mania and bipolar disorder, depression and bipolar disorder

## Abstract

**Purpose:**

Studies of the neural underpinnings of bipolar type I disorder have focused on the emotional control network. However, there is also growing evidence for cerebellar involvement, including abnormal structure, function, and metabolism. Here, we sought to assess functional connectivity of the cerebellar vermis with the cerebrum in bipolar disorder and to assess whether connectivity might depend on mood.

**Methods:**

This cross-sectional study enrolled 128 participants with bipolar type I disorder and 83 control comparison participants who completed a 3 T magnetic resonance imaging (MRI) study, which included anatomical as well as resting state Blood Oxygenation Level Dependent (BOLD) imaging. Functional connectivity of the cerebellar vermis to all other brain regions was assessed. Based on quality control metrics of the fMRI data, 109 participants with bipolar disorder and 79 controls were included in the statistical analysis comparing connectivity of the vermis. In addition, the data was explored for the potential impacts of mood, symptom burden, and medication in those with bipolar disorder.

**Results:**

Functional connectivity between the cerebellar vermis and the cerebrum was found to be aberrant in bipolar disorder. The connectivity of the vermis was found to be greater in bipolar disorder to regions involved in motor control and emotion (trending), while reduced connectivity was observed to a region associated with language production. In the participants with bipolar disorder, past depression symptom burden affected connectivity; however, no effects of medication were observed. Functional connectivity between the cerebellar vermis and all other regions revealed an inverse association with current mood ratings.

**Conclusion:**

Together the findings may suggest that the cerebellum plays a compensatory role in bipolar disorder. The proximity of the cerebellar vermis to the skull may make this region a potential target for treatment with transcranial magnetic stimulation.

## 1. Introduction

Bipolar type I disorder is a major psychiatric disorder with an individual lifetime prevalence of approximately 1.0%. The hallmark feature of bipolar disorder is characterized by mood states (depression and mania) which can be severe and prolonged. Despite being highly heritable (60%–85%) (1), the mechanisms underlying bipolar disorder remain largely unknown. While bipolar disorder has been associated with changes in the emotional control network (2), a potential key region within this network, the cerebellum, has largely been understudied. Classically the cerebellum has been associated with motor control (coordination, timing, and learning), yet the cerebellum is ideally positioned to influence non-motor function including mood regulation. Viral tract tracing studies in non-human primates (3–5) as well as functional imaging studies in humans (6, 7) have identified connections from the cerebellum to non-motor rostral brain structures including those implicated in mood such as the prefrontal cortex, amygdala, basal ganglia, and monoamine producing brainstem nuclei (4, 8–11).

Given these observations of cerebellar connectivity, we were interested in better understanding connectivity of the cerebellum with the cerebrum in bipolar disorder. Resting state functional connectivity employing dynamic Blood Oxygenation Level Dependent (BOLD) imaging provides the unique opportunity to study functional connectivity of neural circuits. To date there have been just a handful of studies that have employed resting state functional connectivity in bipolar disorder to study functional connectivity of the cerebellum and these studies have reported both increased and decreased functional connectivity of the cerebellum with the cerebrum (12–22) with multiple regions involved including the vermis, dentate gyrus, crus II, and flocculus. In addition, Fetah et al. reported differential cerebellar connectivity between bipolar disorder and unipolar depression (17). Of particular interest is the functional connectivity of the cerebellar vermis to the cerebrum due to its high degree of connectivity to nodes involved in emotional regulation including the thalamus (vermis: VIIb and VIIIb) (23), amygdala (vermis: IX) (24), and anterior cingulate cortex (vermis: VI) (23). Thus, the first aim of this study was to explore potential differences in functional connectivity of the cerebellar vermis between bipolar type I disorder (referred to as bipolar disorder throughout) and controls in a relatively large cohort. Our initial hypothesis motivating this work was that the cerebellum serves a compensatory function to brain regions and networks involved in emotional control in bipolar disorder. Thus, we predicted that participants with bipolar disorder would have greater functional connectivity of the cerebellum vermis to the cerebrum when comparing participants with bipolar disorder to controls. Given that functional connectivity associated with mood state in bipolar disorder has just been explored in a small number of studies (25, 26), the second aim of this study was to explore the association of cerebellar functional connectivity with mood ratings of mania and depression. In the analyses looking at the relationship with mood, we hypothesized that functional connectivity of the vermis would be associated with mood such that increased functional connectivity would be related to less severe depressive and mania symptoms suggesting that the cerebellar vermis serves a compensatory role in maintaining normal mood. Finally, we explored if disease burden and medications might have an association with the observed findings.

## 2. Materials and methods

**Figure 1:**
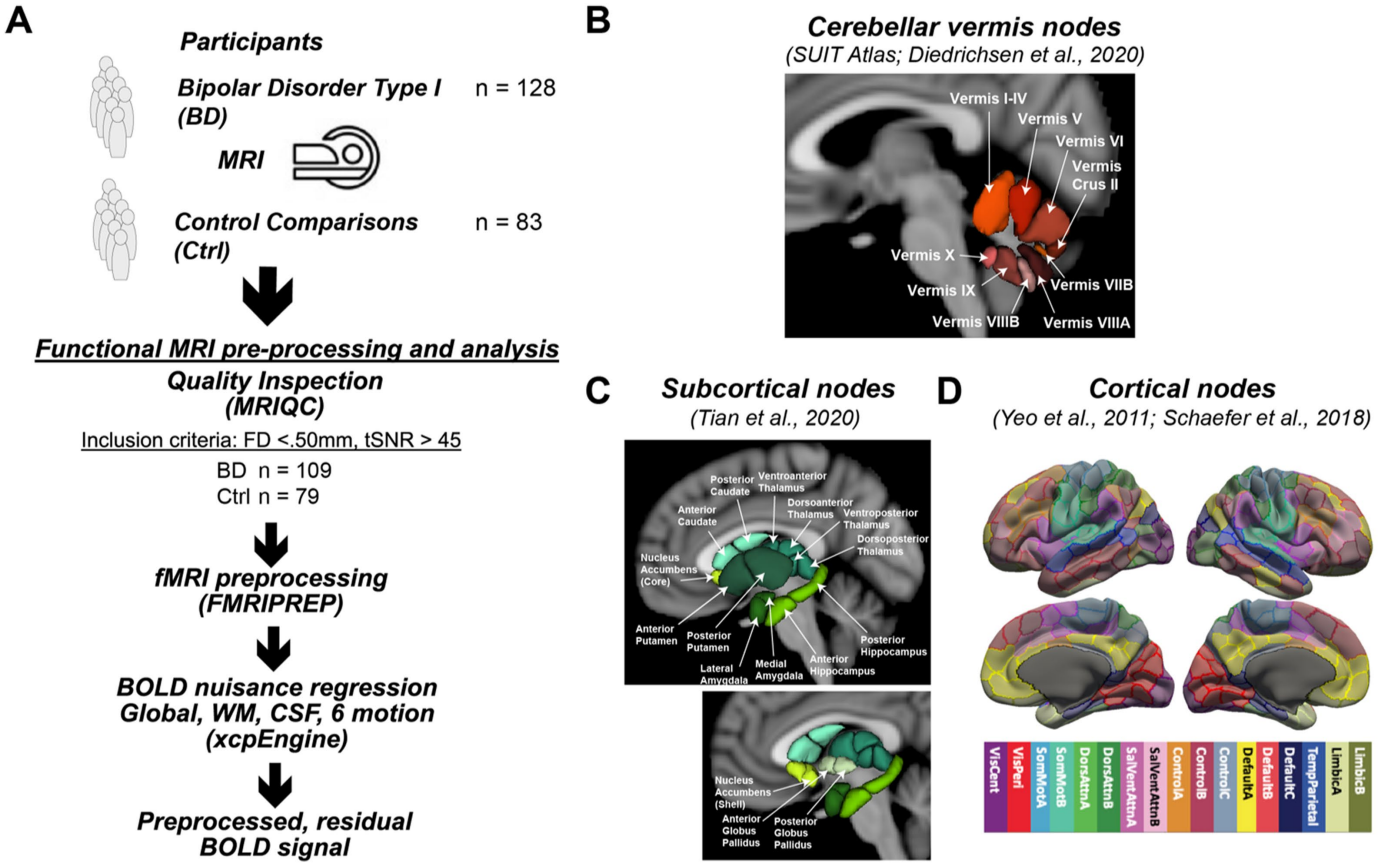
Study design. **(A)** The study enrolled 128 participants with bipolar type I disorder and 83 frequency matched controls into a MR imaging study to assess differences in functional connectivity. Processing of the acquired resting state functional connectivity utilized standard image analysis pipelines including MRIQC and FMRIPREP. After preprocessing, 109 participants with bipolar disorder and 79 controls were used in the statistical analysis. **(B)** Functional connectivity of the cerebellar vermis (SUIT Atlas) with the cerebellum (SUIT Atlas), **(C)** subcortical (Tian Atlas), and **(D)** cortical (Shaefer Atlas) regions was assessed. Specifically, the Schaefer atlas label refers to the Schaefer et al. (27) 400 Parcels at MNI 2 mm resolution, of 17 cortical networks functionally defined by Yeo et al. (28) A detailed map of the parcellation can be seen at the atlas github page (29). The specific image is: Schaefer2018_400Parcels_17Networks_order_FSLMNI152_2mm.nii.gz.

After receiving institutional review board (IRB) approval from the University of Iowa, individuals with bipolar disorder and a frequency matched comparison group were recruited into a cross-sectional neuroimaging study (Figure 1). Potential participants were screened and excluded from the study for comorbid neurological disorders, loss of consciousness for more than 10 min, current drug/alcohol abuse, or MR contraindications. Those who were eligible for the study based on their responses to the screening questions were invited to participate. Participants who provided written informed consent were enrolled into the study and evaluated using the Structured Clinical Interview for DSM Disorders to verify a psychiatric diagnosis of bipolar type I disorder. Control comparison participants were allowed to have prior diagnosis of a mood disorder such as depression as long as they were not receiving active treatment. Additional assessments included the Montgomery-Åsberg Depression Rating Scale (MADRS) (30), Young Mania Rating Scale (YMRS) (31), Adverse Childhood Experiences (ACEs) Questionnaire (32), and the Columbia-Suicide Severity Rating Scale (C-SSRS) (33). Participants also provided a list of current medications. Psychiatric medications were sorted into classes (antidepressants, antipsychotics, sedatives, anticonvulsants, stimulants, and lithium) modified from the WHO Anatomical Therapeutic Chemical System (34). Participants were also asked to retrospectively estimate their symptom burden in terms of what percent of their time was spent in each mood state (depressed, manic, euthymic) over the last decade or since diagnosis (if diagnosis occurred <10 years ago), as previously described (35). The control comparison group was identified by attempted frequency matching for age, sex, race, and social economic status (SES) using the MacArthur Scale of Subjective Social Status (36) of the participants with bipolar disorder. The comparison group was recruited based on advertisements in the local community and a brief screening questionnaire completed by potential participants. At the time of this analysis, the study had enrolled 128 participants with bipolar disorder and 83 control comparison participants who completed an imaging session at 3 T, which included anatomical (T1, T2, and diffusion weighted), resting state functional imaging, and T1ρ imaging. In this report, we focus on the analysis of the resting state functional imaging data collected as part of this project. Most participants also completed a 7 T imaging session focused on metabolic imaging as previously reported in Magnotta et al. (37).

### 2.1. MRI data acquisition

The MRI studies were conducted at the University of Iowa Magnetic Resonance Research Facility (MRRF) on a 3 T General Electric (GE) Discovery MR750W using a 32-channel head coil which was upgraded to a 3 T GE Premier MRI Scanner with a 48-channel head coil. The same imaging protocol was used across scanner configurations. A reference T1-weighted anatomical brain image was collected via a coronal fast spoiled grass sequence (TI = 450 ms, TE = 2.0 ms, TR = 5.2 ms, Flip Angle = 12°, Matrix = 256x256x220, Field of View = 256 × 256 × 220mm, Bandwidth = 488 Hz/pixel) for co-registration of all functional images. Resting state functional images were acquired using an echo-planar gradient-echo sequence with a voxel size of 3.4 × 3.4 × 4mm acquired in an ascending axial slice order and no gap between slices (TE = 30 ms, TR = 2,000 ms, Flip Angle = 80°, Field of View =220x220mm, Matrix = 64 × 64 × 35, Bandwidth = 7,812 Hz/pixel; 300 volumes for a total scan time of 600 s). All participants were instructed to attend their eyes on a fixation cross, relax, and remain awake during the resting state scan.

### 2.2. MRI data preprocessing

All MRI data were converted from DICOM to NIFTI with the dcm2niix software. fMRIPrep (v20.2.0), a Nipype based tool, was then used to preprocess the anatomical and functional images, which included registration with the MNI atlas and nuisance regressor estimation. Several internal operations within fMRIPrep use Nilearn (38) (version 0.6.2, RRID:SCR_001362), mostly within the functional processing workflow. For more details of the pipeline, the reader is referred to the workflows section in the fMRIPrep documentation.

#### 2.2.1. Anatomical data preprocessing

The T1-weighted (T1w) image was corrected for intensity non-uniformity using the N4 bias field correction algorithm (39) (N4BiasFieldCorrection) distributed with ANTs (40) (version 2.3.3, RRID:SCR_004757). The resulting bias field corrected T1 weighted (T1w) image was then subsequently used as the T1w-reference image throughout the remainder of the workflow. The T1w-reference image was then skull-stripped using the ANTS based brain extraction workflow (antsBrainExtraction.sh) with the OASIS30ANTs atlas used as the target template. Tissue classification was then performed on the brain-extracted T1w-reference image using FSL (41) (fast version 5.0.9, RRID:SCR_002823) to label each voxel as cerebrospinal fluid, white matter, or gray matter. Next, brain surfaces were reconstructed using FreeSurfer (42) (recon-all version 6.0.1, RRID:SCR_001847), and the brain mask was refined by employing a custom variation of the method to reconcile ANTs and FreeSurfer derived segmentations of the cortical gray matter using Mindboggle (43) (RRID:SCR_002438). Volume-based spatial normalization to the MNI atlas (version MNI152NLin2009cAsym) was performed through a nonlinear registration with ANTs (44) (antsRegistration), using brain-extracted versions of both the T1w-reference and the T1 image from the MNI Atlas.

#### 2.2.2. Functional data preprocessing

For the 10 min of resting state BOLD data collected per participant, the following preprocessing steps were performed. First, a reference volume and its skull-stripped version were generated based on the median volume in the BOLD timeseries, without susceptibility distortion correction. The BOLD reference volume was then co-registered to the skull-stripped T1w reference using boundary-based registration in FreeSurfer (45) (bbregister). Time series co-registration was then performed by concatenating transforms from (1) the rigid body realignment parameters, (2) affine and boundary-based registration of the BOLD reference image to T1w-reference image, and (3) non-linear registration of the T1w-reference image to MNI space. The resulting concatenated transform was used to transform the BOLD time series into MNI space using a single step of interpolation with a Lanczos kernel. The resulting rotation/translation parameters along with the framewise displacement (FD) were saved for use as nuisance regressors as well as quality assurance metrics, respectively, (46).

Functional MRI data from participants was considered eligible for further analyses if the mean framewise displacement was below 0.50 mm, and the temporal signal-to-noise ratio (tSNR) was greater than 45. This approach resulted in 23 participants being excluded (*n* = 19 bipolar disorder, n = 4 controls). In all, 109 bipolar disorder participants and 79 control participants were included in the subsequent analyses evaluating the connectivity of the cerebellar vermis.

With the functional images in the MNI coordinate system, functional connectivity was assessed by combining three atlases to provide regions of interest, which included cortical brain regions from the Schaefer 400 atlas parcellated into 17 networks (27), sub-cortical regions from the Tian atlas at Scale II (47), and cerebellar regions from the SUIT atlas (48). A modified version of the SUIT atlas was used in this study since only cerebellar vermal regions defining lobules VI-X are included in the atlas. To define the anterior lobe portion of the vermis (I-V), the vermal regions for this portion of the cerebellum (I-IV and V) were defined as the medial portions of these lobules with the same width as vermis VI. As a result, the full set of vermal ROIs included sub-regions I-IV, V, VI, Crus II, VIIB, VIIIA, VIIIB, IX, and X (Figure 1B). The number of voxels of the cerebellar ROIs at the resolution of the acquired fMRI data was as follows: I-IV (66 voxels), V (5 voxels), VI (56 voxels), Crus II (7 voxels), VIIB (1 voxel), VIIIA (23 voxels), VIIIB (13 voxels), IX (15 voxels), and X (3 voxels). The 445 regions without signal dropout were then subsequently used in the functional connectivity analysis as described in the following sections.

### 2.3. Functional connectivity analyses

#### 2.3.1. Denoising

Steps were taken to mitigate the influence of artifacts on resting state BOLD time series before assessing functional connectivity between regions. This included nuisance regression of the average signal from the participant-specific segmentations of white matter and ventricles, the global signal, as well as the 6 rigid body realignment parameters (3 translational, 3 rotational) using a modified xcpEngine design file (49) (version 1.2.4). Temporal filtering was conducted during the nuisance regression step using AFNI (50) (3dBandpass, Butterworth filter), which ensures BOLD fluctuations are within the frequency band of 0.01 < *f* < 0.08 Hz. The output of this regression based denoising is a residualized BOLD timeseries that is mean-centered at zero.

#### 2.3.2. Cerebellar vermal functional connectivity

Following preprocessing, the residualized BOLD timeseries was extracted from each of the brain regions defined in the combined brain atlas described previously. The mean time series of voxels within each atlas region was used to estimate the functional connectivity by computing the Pearson’s Correlation between each vermal ROI and all ROIs included in the combined brain parcellation scheme. The resulting correlation coefficient (r) was then converted into a Fisher’s Z [i.e., z(r)]. Given the large number of comparisons that would result from considering the connections between all ROI pairs, we limited the connections of interest to those between the defined cerebellar vermal ROIs and all other regions. In addition, connections between the vermal ROIs that had a negative mean correlation coefficient for both groups of participants were excluded from further analyses.

### 2.4. Statistical analyses

For each vermal ROI, linear regression analyses were run in R/RStudio version 4.1.1 (51, 52) with packages tidyverse (53), broom (54), and arsenal (55) for all possible connections with other brain regions that survived the constraints described above. The primary analysis for this study was to compare resting state functional connectivity between participant groups (bipolar disorder vs. control comparison). For this analysis, the linear models contained diagnosis as the independent variable of interest as well as covariates to control for age, sex, and tSNR with functional connectivity between the vermal ROIs and all other regions in the brain used as the dependent variables (each connection was run as a separate regression model). To account for multiple comparisons in this mass univariate approach, *p*-values were adjusted using false discovery rate (FDR) separately for each vermal ROI. Adjusted *p*-values (q-values) were considered significant at q < 0.05. However, since screening for positive connectivity resulted in a different number of statistical tests evaluated per vermal ROI (between 178 and 257; see Supplementary Table 1), we have also reported all sites with raw uncorrected *p*-values < 0.001, which we refer to as trending findings. To visualize the relative contribution and direction of diagnosis to the estimated difference in functional connectivity between groups, we calculated the partial residuals using the jtools package (56) of the corresponding linear regression model.

Follow-up analyses were conducted in only the participants with bipolar disorder for the vermal connections with a between group difference (bipolar disorder vs. control) meeting the *p* < 0.001 threshold. For these analyses, linear models were again used to assess whether past symptom burden, current mood, or current medications might be contributing to the significant or trending effects. The linear models also included covariates for age, sex, and tSNR. For past symptom burden ratings, we used k-means clustering to identify “low” and “high” groups, as the distributions appeared bimodal, and this binary variable was used in the models. In evaluating the association between current mood and functional connectivity, MADRS or YMRS total scores were used as continuous independent variables of interest in the linear models. For the medication analysis, separate models were used for each class of drugs where a binary variable (0 = off, 1 = on) was used to code if a participant was on a particular class of medications. In these models, medication class was the variable of interest in the linear model with the same covariates used as in the other linear models.

Finally, additional exploratory analyses were conducted to explore the relationship of current mood, symptom burden, or current medications on vermal connectivity in participants with bipolar disorder across all regions using the same linear models described previously. FDR correction was used to correct for multiple comparisons, with q < 0.05 considered significant.

In reporting the results, the significant/trending ROI pairs for which the target was part of the Schaefer Cortical Atlas (27), the common name of the target was identified by matching the MNI coordinates of the ROI’s center of mass with the Harvard-Oxford Cortical Structural Atlas provided within the FSL package. As this atlas provides the percent likelihood that a given coordinate is part of a specific anatomical structure, the structure with the highest percentage match was selected as the common name reported in the figures and tables.

## 3. Results

The demographics for the participants whose resting state functional connectivity data was used in the linear models is summarized in Table 1. The two groups (bipolar disorder and comparison control) were well balanced for sex with each group containing approximately a 2:1 ratio of females to males. The groups had similar ages and age ranges with the mean age of the participants being 39 years of age with participants ranging from 18 to 70 years of age. Participants with bipolar disorder and comparison controls also had similar educational attainment of 15 years of school, but the controls did report significantly higher perceived social economic status of 1 rung on the MacArthur Scale (6.2 for comparison controls vs. 5.3 for bipolar disorder). As expected, the participants with bipolar disorder had significantly higher mood ratings for both mania (YMRS total scores: 6.0 vs. 0.9) and depression (MADRS total scores: 14.4 vs. 2.8) as compared to the controls. They also had significantly more adverse childhood events and more suicide attempts as compared to the controls with 2 control participants having a prior suicide attempt and 50 of the participants with bipolar disorder having a prior attempt.

**Table 1:** Participant demographics.

Variable	Control (*N* = 79)	Bipolar I (*N* = 109)	Test statistic	*p*-Value
Sex (males, females)	(27, 52)	(41, 68)	0.234^†^	0.628
Age (years; mean ± SD)	39.8 ± 14.0	38.5 ± 13.2	−0.598^††^	0.550
Age range (years)	18–70	18–66		
Educational attainment (years)	15.4 ± 2.09	15.0 ± 2.11	−1.268^††^	0.205
Self-reported SES^‡^	6.24 ± 1.30	5.29 ± 1.96	−3.446^††^	<0.001^*^
ACE score	1.44 ± 1.84	3.52 ± 2.60	5.272^††^	<0.001^*^
YMRS score	0.92 ± 1.67	5.97 ± 5.99	7.654^††^	<0.001^*^
MADRS score	2.81 ± 4.52	14.4 ± 9.75	8.902^††^	<0.001^*^
Suicide attempts	0.03 ± 0.16	1.68 ± 3.93	6.639^††^	<0.001^*^
% time depressed^‡‡^		38.02 ± 25.87		
% time manic^‡‡^		18.89 ± 17.55		
Medication class (on, off)				
Antidepressants		(59, 50)		
Antipsychotics		(59, 50)		
Sedatives		(44, 65)		
Anticonvulsants		(45, 64)		
Lithium		(30, 79)		
Stimulants		(10, 99)		

† Chi-squared test or ^††^Wilcoxon-Mann–Whitney converted to Z-score, as appropriate.

‡ MacArthur Scale of Subjective Social Status.

‡‡ Self-reported, considering the past 10 years or since onset if < 10 years.

* Value of *p* < 0.05.

### 3.1. Functional connectivity differences between bipolar disorder and controls

**Figure 2:**
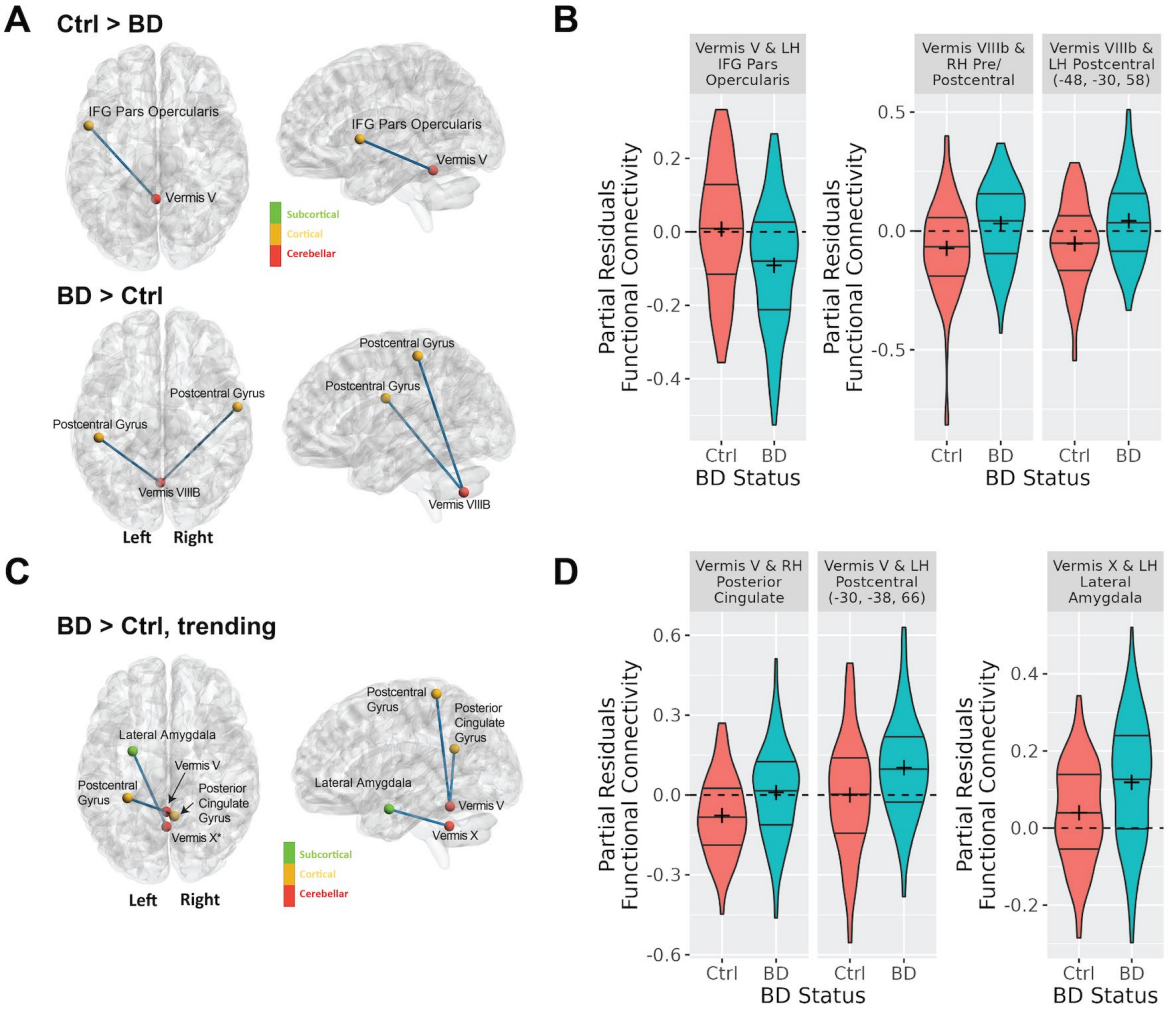
Differences in cerebellar vermal connectivity in bipolar type I disorder as compared to controls identified using a linear regression analysis including covariates for age, sex, tSNR. Panel **(A)** shows connections with significantly (FDR corrected q-value < 0.05) different connectivity in bipolar disorder as compared to controls. Panel **(B)** shows the functional connectivity for the bipolar and control groups for the connections shown in **(A)**. This data shows the partial residuals after controlling for age, sex, and tSNR. Panel **(C)** shows connections with trending (defined as uncorrected *p-*value < 0.001) differences connectivity differences in bipolar disorder as compared to controls. Panel **(D)** shows the functional connectivity for the bipolar and control groups for the connections shown in **(C)**. The data shown are the partial residuals after controlling for age, sex, and tSNR.

Supplementary Table 1 shows the number of comparisons that were considered in the analysis for each vermal node after removing those regions with a negative mean correlation coefficient for both groups of participants as outlined in section 2.3.2. When initiating this study our hypothesis was that functional connectivity between the cerebellar vermis and other brain regions would differ between participants with bipolar disorder and controls. Supporting this hypothesis, a linear regression analysis for main effect of diagnosis revealed significant differences (FDR; q < 0.05) in vermal connectivity between participants with bipolar disorder vs. controls for the following three connections (see Figure 2; Supplementary Figure 1 (raw Fisher-Normalized Z-scores), and Table 2): vermis lobule V to left pars opercularis (Schaefer network SalVentAttnA), vermis lobule VIIIB to left postcentral gyrus (Schaefer network SomMotA), and vermis lobe VIIIB to right pre/postcentral gyrus (Schaefer network SomMotB). Additionally, trending differences (*p* < 0.001) in vermal connectivity were also identified between participants with bipolar disorder and controls (also shown in Figure 2; Table 2): vermis lobule V to right posterior cingulate gyrus (Schaefer network DefaultA), vermis lobule V to left postcentral gyrus (Schaefer network SomMotA), and vermis lobule X to left lateral amygdala. With the exception of the connectivity between the vermis lobule V and left pars opercularis, the participants with bipolar disorder had a higher functional connectivity for all significant and trending differences in vermal connectivity as evident by the positive Beta coefficients estimated from the linear models.

**Table 2:** Regression analysis showing the effect of group (bipolar-controls) on functional connectivity (Pearson’s correlation) of the cerebellar vermis.

Seed name and MNI coordinate	Target common name MNI coordinate	Atlas	Atlas label name (network or region)	*p*-value	q-value	Beta coefficient
Vermis V (0, −48.5, −20)	Left pars opercularis (−52, 8, 14)	Schaefer	SalVentAttnA	0.0002	0.036^*^	−0.099
	Right posterior cingulate gyrus (6, −52, 24)	Schaefer	DefaultA	0.0007	0.071	0.085
	Left postcentral gyrus (−30, −38, 66)	Schaefer	SomMotA	0.0009	0.071	0.102
Vermis VIIIB (0, −65, −45)	Right pre/postcentral gyrus (60, −6, 26)	Schaefer	SomMotB	0.0002	0.040^*^	0.105
	Left postcentral gyrus (−48, −30, 58)	Schaefer	SomMotA	0.0003	0.040^*^	0.096
Vermis X (0, −48, −35)	Left lateral amygdala (−26, −2, −22)	Tian	Left lateral amygdala	0.0005	0.096	0.079

All results with an uncorrected *p*-value < 0.001 are shown along with their false discovery rate q-value and regression beta coefficient for the effect of group. The regression analysis included covariates for age, sex, and tSNR.

* False Discovery Rate q-value < 0.05.

Next, we sought to examine factors that may be influencing variability in the bipolar group in the six identified significant or trending ROI pairs. We started by examining the association of current mood ratings of depression (MADRS) and mania (YMRS) with functional connectivity of the cerebellar vermis. MADRS score was positively associated with increased connectivity between vermis lobule V and vermis lobule VIIIB to two nodes in the left postcentral gyrus, and negatively associated with connectivity between vermis lobule V and the right posterior cingulate gyrus (Table 3). YMRS score was not associated with connectivity in any of the identified ROI pairs.

**Table 3:** In participants with bipolar disorder, current MADRS ratings significantly correlated with differences in connectivity for the sites found to be different or trending by diagnosis (see Table 2; Figure 2).

Model type	Seed name and MNI coordinate	Target common name and MNI coordinate	Atlas	Atlas label name (network or region)	*p*-value	Beta coeff.
MADRS	Vermis V (0, −48.5, −20)	Left IFG pars opercularis (−52, 8, 14)	Schaefer	SalVentAttnA	0.630	0.0008
		Right posterior cingulate gyrus (6, −52, 24)	Schaefer	DefaultA	0.020*	−0.004
		Left postcentral gyrus (−30, −38, 66)	Schaefer	SomMotA	0.043*	0.004
	Vermis VIIIB (0, −65, −45)	Right pre/postcentral gyrus (60, −6, 26)	Schaefer	SomMotB	0.093	0.003
		Left postcentral gyrus (−48, −30, 58)	Schaefer	SomMotA	0.033^*^	0.004
	Vermis X (0, −48, −35)	Left lateral amygdala (−26, −2, −22)	Tian	Left lateral amygdala	0.827	−0.0004
YMRS	Vermis V (0, −48.5, −20)	Left IFG pars opercularis (−52, 8, 14)	Schaefer	SalVentAttnA	0.288	−0.003
		Right posterior cingulate gyrus (6, −52, 24)	Schaefer	DefaultA	0.440	−0.002
		Left postcentral gyrus (−30, −38, 66)	Schaefer	SomMotA	0.929	−0.0003
	Vermis VIIIB (0, −65, −45)	Right pre/postcentral gyrus (60, −6, 26)	Schaefer	SomMotB	0.449	0.002
		Left postcentral gyrus (−48, −30, 58)	Schaefer	SomMotA	0.975	−0.00009
	Vermis X (0, −48, −35)	Left lateral amygdala (−26, −2, −22)	Tian	Left lateral amygdala	0.923	0.0003

Current YMRS ratings did not significantly correlate with connectivity for any of the 6 sites. All models used sex, age, and tSNR as covariates.

Because previous studies have suggested that illness severity (e.g., number of episodes) may be linked to changes in cerebellar structure in bipolar disorder (57–60), we examined whether 10-year (or since diagnosis) symptom burden significantly contributed to functional connectivity in the significant and trending ROI pairs identified in Table 2. Participant self-ratings were grouped into “high” and “low” using k-means clustering. Within participants with bipolar disorder, higher depression symptom burden was associated with increased connectivity between vermis lobule VIIIb and left postcentral gyrus and vermis VIIIb and right pre/postcentral gyrus, and reduced connectivity between vermis lobule V and right posterior cingulate (Table 4). Mania symptom burden ratings did not correlate with connectivity for any of the ROI pairs (Table 4).

**Table 4:** In participants with bipolar disorder, association between past 10-year symptom burden (or since diagnosis) and vermal connectivity identified as different between participants with bipolar disorder and controls (Table 2).

Model type^†^	Seed name and MNI coordinate	Target common name and MNI coordinate	Atlas	Atlas label name (network or region)	*p*-value	Beta coeff.
Past depression symptom burden (low vs. high)	Vermis V (0, −48.5, −20)	Left IFG pars opercularis (−52, 8, 14)	Schaefer	SalVentAttnA	0.439	0.027
		Right posterior cingulate gyrus (6, −52, 24)	Schaefer	DefaultA	0.0468^*^	−0.071
		Left postcentral gyrus (−30, −38, 66)	Schaefer	SomMotA	0.462	0.029
	Vermis VIIIB (0, −65, −45)	Right pre/postcentral gyrus (60, −6, 26)	Schaefer	SomMotB	0.041^*^	0.070
		Left postcentral gyrus (−48, −30, 58)	Schaefer	SomMotA	0.003^*^	0.102
	Vermis X (0, −48, −35)	Left lateral amygdala (−26, −2, −22)	Tian	Left lateral amygdala	0.863	0.006
Past mania symptom burden (low vs. high)	Vermis V (0, −48.5, −20)	Left IFG pars opercularis (−52, 8, 14)	Schaefer	SalVentAttnA	0.805	−0.009
		Right posterior cingulate gyrus (6, −52, 24)	Schaefer	DefaultA	0.156	−0.050
		Left postcentral gyrus (−30, −38, 66)	Schaefer	SomMotA	0.383	0.033
	Vermis VIIIB (0, −65, −45)	Right pre/postcentral gyrus (60, −6, 26)	Schaefer	SomMotB	0.792	0.009
		Left postcentral gyrus (−48, −30, 58)	Schaefer	SomMotA	0.849	0.007
	Vermis X (0, −48, −35)	Left lateral amygdala (−26, −2, −22)	Tian	Left lateral amygdala	0.742	−0.011

All models used sex, age, and tSNR as covariates.

* *p* < 0.05.

† Symptom burden was divided between low and high based on k-means clustering.

Linear regression models were also used to explore the effects of medication in the participants with bipolar disorder in the identified ROI pairs from Table 2. In these analyses, medications were grouped into different classes and those with sufficient sample size defined as at least 25% of the sample size (i.e., at least 27 subjects) in each group (on and off medication) were included in separate regression models. This allowed us to explore the effects of the following medications: lithium, antipsychotics, antidepressants, anticonvulsants, and sedatives on the findings reported above. The results showed no significant effect of medication on the results even before correction for multiple comparisons (Supplementary Table 2). Thus, it is does not appear that medication contributed to the effects of bipolar disorder that were observed in the primary analysis.

### 3.2. Exploratory analyses of vermal functional connectivity with all brain regions associated with mood in bipolar disorder

**Figure 3:**
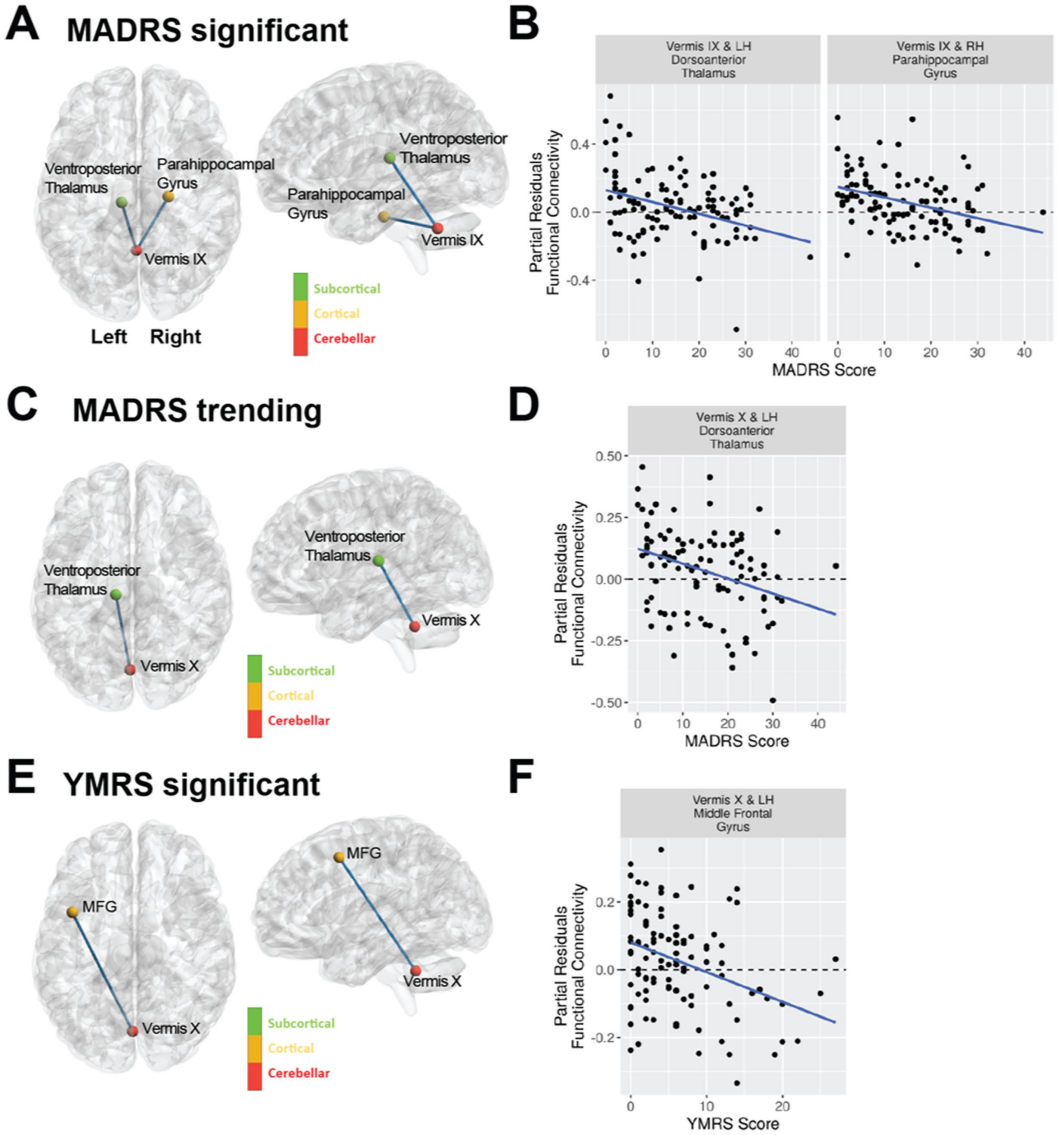
Mood as assessed with the MADRS and YMRS scales is associated with vermal connectivity in bipolar type I disorder using a regression analysis including covariates for age, sex, and tSNR. The violin plots shown are the partial residuals after controlling for age, sex, and tSNR. **(A)** Cerebellar vermal functional connectivity with a significant (FDR corrected q-value < 0.05) association with MADRS total scores. Panel **(B)** shows the regression between MADRS and functional connectivity for the regions identified in **(A)**. **(C)** Cerebellar vermal functional connectivity with trending (uncorrected *p-*value < 0.001) association with MADRS total scores. Panel **(D)** shows the regression between MADRS and functional connectivity for the regions identified in **(C)**. **(E)** Cerebellar vermal functional connectivity with a significant (FDR corrected q-value < 0.05) association with YMRS total scores. Panel **(F)** shows the regression between YMRS and functional connectivity for the regions identified in **(E)**.

While we had examined the effects of mood in bipolar disorder for the vermal connections with significant or trending differences between diagnostic groups, we wondered whether mood could be playing a significant role in vermal connectivity with other regions. Thus, we conducted an exploratory analysis with FDR correction across all ROI pairs for each vermal lobule. The significant results of the statistical models are summarized in Figure 3, Table 5, and Supplemental Figure 2. Total scores from the MADRS scale were significantly (FDR; q < 0.05) associated with functional connectivity of vermis lobule IX to left ventroposterior thalamus and vermis lobule IX to right anterior parahippocampal gyrus (Schaefer network DefaultC). In addition, there was a trending (*p* < 0.001) association with functional connectivity of the vermis lobule X to left ventroposterior thalamus. The direction for all of the significant and trending associations with MADRS scores was negative, such that higher functional connectivity values were associated with lower MADRS scores. Looking at the relationship between YMRS scores and functional connectivity, linear regression found a significant association (FDR; q < 0.05) between vermis lobule X and left middle frontal gyrus (Schaefer network DefaultB). As with the MADRS analysis, the direction of the association was negative, such that higher functional connectivity values were associated with lower YMRS scores.

**Table 5:** Functional connectivity of the cerebellar vermis and association with mood in bipolar disorder.

Model	Seed name (MNI coordinate)	Target common name (MNI coordinate)	Atlas	Atlas label name (network/region)	*p*-value	q-value	Beta coefficient
MADRS	Vermis IX (0, −55, −36)	Left dorsoanterior thalamus (−10, −22, 12)	Tian	Left ventroposterior thalamus	0.0004	0.038^*^	−0.007
	Vermis IX (0, −55, −36)	Right parahippocampal gyrus (22, −18, −28)	Schaefer	DefaultC	0.0001	0.025^*^	−0.006
	Vermis X (0, −48, −35)	Left dorsoanterior thalamus (−10, −22, 12)	Tian	Left ventroposterior thalamus	0.0005	0.108	−0.006
YMRS	Vermis X (0, −48, −35)	Left middle frontal gyrus (−42, 8, 48)	Schaefer	DefaultB	0.0001	0.025^*^	−0.009

All connections with a *p*-value < 0.001 are shown along with the corresponding FDR q-value and regression beta coefficient for the effect of mood ratings. The regression analysis included covariates for age, sex, and tSNR.

* False discovery rate q-value < 0.05.

### 3.3. Exploratory analyses of vermal functional connectivity with all brain regions associated with symptom burden in bipolar disorder

As with mood, we considered whether symptom burden ratings might be associated with connectivity of the vermis to any other brain region (Table 6). We found that higher past depression symptom burden was significantly associated with increased connectivity of vermis lobule VIIB to the insular cortex and central opercular cortex (Schaefer network SalVenAttnA). There were also trends (*p* < 0.001) for higher past depression to associate with increased connectivity between Vermis VIIIB and the precentral gyrus (Schaefer network SomMotA) as well as Vermis lobule IX to the superior frontal gyrus (Schaefer network SalVentAttnA). No vermal nodes survived FDR correction for past mania symptom burden, but there were trends (*p* < 0.001) for associations between higher mania burden and increased connectivity between vermis lobule V and the intracalcarine sulcus (Schaefer network VisPeri) and decreased connectivity between vermis lobule V and temporal pole (Schaefer network TempPar) as well as vermis crus II to lateral occipital cortex (Schaefer network DorsAttnA).

**Table 6:** In participants with bipolar disorder, association between past 10-year symptom burden (or since diagnosis) and vermal connectivity to all brain regions.

Model type^†^	Seed name and MNI coordinate	Target common name and MNI coordinate	Atlas	Atlas label name (network or region)	*p*-value	q-value	Beta coeff. (effect of higher burden)
Past depression symptom burden (low vs. high)	Vermis VIIB (1, −69, −31)	Insular cortex (−38, 2, −4)	Schaefer	SalVentAttnA	0.0003	0.029^*^	0.117
		Central opercular cortex (−50, 2, 4)	Schaefer	SalVentAttnA	0.0002	0.029^*^	0.105
	Vermis VIIIB (0, −65, −45)	Precentral gyrus (38, −20, 64)	Schaefer	SomMotA	0.0006	0.105	0.122
	Vermis IX (0, −55, −36)	Superior frontal gyrus (6, 10, 58)	Schaefer	SalVentAttnA	0.0006	0.112	0.113
Past mania symptom burden (low vs. high)	Vermis V (0, −48.5, −20)	Intracalcarine sulcus (−18, 64, 6)	Schaefer	VisPeri (visual peripheral)	0.0004	0.059	0.137
		Temporal pole (48, 16, −20)	Schaefer	TempPar (temporo-parietal)	0.0008	0.059	−0.145
	Vermis crus II (1, −74, −32)	Lateral occipital cortex, inferior division (48, −66, 4)	Schaefer	DorsAttnA	0.0003	0.064	−0.131

All connections with a *p*-value < 0.001 are shown along with the corresponding FDR q-value and regression beta coefficient for the effect disease burden. The regression analysis included covariates for age, sex, and tSNR. ^†^Symptom burden was divided between low and high based on k-means clustering.

* False Discovery Rate q-value < 0.05.

### 3.4. Exploratory analyses of medication impact on whole brain vermal functional connectivity in bipolar disorder

We also conducted an exploratory analysis of the effects of medication class on all the connections of the cerebellar vermis in participants with bipolar disorder (Table 7). The resulting models found a significant effect for antidepressant medications increasing functional connectivity strength between the vermis lobule I-IV to the left posterior globus pallidus and left orbital frontal cortex (Schaefer network DefaultB). There was also a trend (*p* < 0.001) for lithium to increase functional connectivity between vermis lobule VIIB and the left posterior parahippocampal gyrus (Schaefer network DefaultC) as well as the vermis lobule VIIA to left precentral gyrus (Schaefer network SomMotB).

**Table 7:** Regression analysis evaluating functional connectivity in participants with bipolar disorder on and off various classes of medications evaluating the effect of all cerebellar vermis connections.

Medication class	Seed name and MNI coordinate	Target common name and MNI coordinate	Atlas	Atlas label name (network or region)	*p*-value	q-value	Beta value
Antidepressants	Vermis I-IV (0, −45.5, −16.5)	Left posterior globus (−22, −8, −2)	Tian	Left posterior globus pallidus	0.0003	0.048^*^	0.145
	Vermis I-IV (0, −45.5, −16.5)	Left orbitofrontal cortex (−36, 22, −16)	Schaefer	DefaultB	0.0005	0.048^*^	0.139
Antipsychotics	--	--	--	--	--	--	--
Sedatives	--	--	--	--	--	--	--
Anticonvulsants	--	--	--	--	--	--	--
Lithium	Vermis VIIb (1, −69, −31)	Left posterior parahippocampal gyrus (−30, −32, −18)	Schaefer	DefaultC	0.0005	0.103	0.122
	Vermis VIIIa (1, −70, −42)	Left precentral gyrus (−60, −2, 10)	Schaefer	SomMotB	0.0007	0.190	0.136

Covariates for age, sex, and tSNR were included in the model. All cerebellar vermis connections with a *p*-value < 0.001 for the effect of medication class are shown along with the corresponding FDR corrected q-value and estimated beta coefficient for the effect of medication.

* False discovery rate q-value < 0.05.

## 4. Discussion

In this study, we observed differences in functional connectivity of the cerebellar vermis with regions associated with motor function, language, and emotion (trending) in participants with bipolar disorder as compared to a matched control sample. In participants with bipolar disorder, connectivity between vermal nodes and the motor-and emotion-related identified ROIs was associated with current depression severity or past-decade depression symptom burden, but not current mania severity, past-decade mania symptom burden, or medications. Exploratory analyses revealed other vermal connections that were associated with current mood, past symptom burden, and medication in bipolar disorder, with the majority of these connections associated with default-and attention-related functional networks (27). Taken together, these results suggest that vermal functional connectivity is altered in bipolar disorder and may be sensitive to current and past depression symptoms.

The sensorimotor connections with the vermis where differences were observed between bipolar disorder and controls included precentral and postcentral gyrus. The participants with bipolar disorder exhibited greater connectivity between the cerebellar vermis and these sensorimotor regions as compared to controls. There have been some previous reports of subtle abnormal motor functioning in bipolar disorder (61–65). One potential interpretation of this data could be that sensory motor regions are receiving too much input from the cerebellum, specifically from vermal lobules V and VIIIB. Interestingly, both current depression severity and past depression symptom burden were associated with increased functional connectivity in these vermal to sensorimotor connections, which suggests that this elevated connectivity may be mood-state specific or related to overall disease severity.

Connectivity of the cerebellar vermis was also associated with decreased connectivity to the left pars opercularis in the participants with bipolar disorder. This is a region known to be involved in language production. Individuals with bipolar disorder especially during mania often exhibit pressured speech suggesting potential lack of inhibition of the vermis to the pars opercularis. There may also be advantages to disinhibition. The seminal work by Nancy Andreasen and colleagues on creativity and psychiatric disorders (66, 67) suggests bipolar disorder is common in creative writers. It is tempting to speculate that a loss of inhibition from cerebellum to language areas might increase free flow of loosely related words, ideas, and concepts and thus promote creative expression.

The vermal connections where trending differences were observed between bipolar disorder and controls included regions involved in emotional regulation including the amygdala and posterior cingulate. Both of these connections were increased in participants with bipolar disorder as compared to the controls thus suggesting a potential compensatory role for the cerebellum associated with emotional regulation. Consistent with possible compensation during euthymia, connectivity between vermis lobule V and the posterior cingulate was reduced with increasing MADRS score among participants with bipolar disorder. Furthermore, exploratory analyses of mood ratings revealed that vermis connectivity to regions involved in emotional salience (right anterior parahippocampal gyrus) (68, 69) and regulation (left middle frontal gyrus) (70) were associated with reduced connectivity in participants with bipolar disorder who reported more severe mood ratings. The identified regions in the cerebrum have been previously reported to be involved in processing of emotional faces (71) as well as being abnormal when comparing the blood flow response in participants with bipolar disorder to controls using an emotional face paradigm (72–74). Interestingly, the vermal connections to the cerebrum associated with depression (left thalamus and right parahippocampal gyrus) were different from those associated with mania (left middle frontal gyrus). All of the significant relationships with mood to regions involved with emotion were such that increased functional connectivity was associated with less severe mood ratings. Together these findings suggest the possibility that different mood states may result from the vermis failing to serve a compensatory role with different regions of the brain associated with emotion. The reason for this is still unknown but differences in cerebellar metabolism in bipolar disorder could be one potential explanation and an observation which we have found in a sub-sample of these participants as previously reported (37).

Several prior studies have observed differences in functional connectivity of the cerebellum in bipolar disorder. These studies include several that have observed a similar pattern of abnormal connectivity as observed in this study. For example, Olivito et al. found increased connectivity between the deep cerebellar (dentate) nuclei to the amygdala and posterior cingulate in participants with bipolar disorder type 1 as compared to controls (20). Similarly, Wang et al. observed increased long and short-range functional connectivity in the cerebellum in participants with bipolar disorder (75). Furthermore, Li et al. observed decreased functional connectivity of the amygdala with the cerebellum in participants with bipolar disorder during mania and depression (19). Interestingly, individuals diagnosis with bipolar disorder type II (13, 20, 22) and major depression disorder (20) in these studies were found to have a different pattern of cerebellar connectivity that was often in the opposite direction of the findings observed here. Finally, some studies have reported functional connectivity in the opposite direction as those observed here. Li et al. found decreased functional connectivity of the cerebellar vermis to the ventral prefrontal cortex and middle cingulate cortex (18). Similarly Bellani et al. found decreased functional connectivity of cerebellar resting state networks (12) and Chrobak at el. found decreased connectivity of the cerebellum to frontal eye fields and thalamus in bipolar disorder (14). In addition, Shinn et al. observed decreased cerebellar functional connectivity to several resting state networks in bipolar disorder with psychosis (21). It should also be noted that it has been shown that cerebellar connectivity can be modulated by treatment (16), which we also observed for the cerebellar vermis in our exploratory medication analyses. Additionally, based on our findings for past-decade depression symptom burden, it appears that disease burden (self-reported symptom burden) may modulate cerebellar connectivity. Given the variation in the subject populations evaluated across studies it is not surprising that there is variation in the observed findings.

Development of the cerebellum starts approximately 5 weeks post-conception and extends until 2 years postnatal also making it vulnerable to environmental perturbations both *in utero* as well as postnatal. One frequently reported environmental influence is adverse childhood events which occur during this critical time of brain development. As expected based on prior literature, the participants with bipolar disorder in this study exhibited a significantly greater number of adverse childhood events as compared to the controls. However, given that the measure was self-reported it is unlikely that any of the individuals were able to report events from this window of development. The importance of the vermis related to mood regulation has also been previously observed. A prior study reported that a lesion to the posterior vermis was associated with the development of mania as well as changes in functional connectivity to the prefrontal cortex, striatum, and postcentral gyrus (76). Furthermore, the vermis has been implicated in the development of psychotic features of bipolar disorder, in particular hallucinations, using a lesion network mapping approach (77).

In this study we did not observe any associations between medication and the primary outcomes. However, in an exploratory analysis of this data assessing all vermal connections a significant effect of medication was observed for antidepressants and lithium. Each association with lithium was in the direction of increased connectivity for individuals on this medication, resulting in functional connectivity more similar to the comparison control sample, but this was not tested inferentially. Most of the medication studies in participants with bipolar disorder that have assessed changes in functional connectivity have been related to treatment with lithium. A prior study by Altinay et al. found that 8 weeks of treatment increased functional connectivity of the amygdala and medial orbital frontal cortex. The increased connectivity as a result of treatment provided similar connectivity as compared to a comparison control sample (78). Another study by this same group also found that treatment with lithium normalized functional connectivity clustering coefficient by decreasing this measure during mania/hypo-mania and increasing this measure during depression (79). The effects of antidepressants on resting state functional connectivity have not been studied in bipolar disorder. Nearly all of the participants with bipolar disorder in this cross-sectional study were on treatment at the time of this study with many receiving multiple classes of medication. Thus, it is difficult to fully disentangle the treatment effects especially if there are medication interactions.

While this study was one of the larger imaging studies in bipolar disorder containing 109 participants with bipolar type I disorder in the final analysis along with 79 controls, a number of limitations exist in the study. First, the study was cross-sectional in nature and the associations with mood can be only inferred based on the mood of an individual at the time they were assessed. Thus, we were not able to assess fluctuations in functional connectivity within individuals as their mood fluctuated. In the future, longitudinal studies following subjects across various mood states would help to better understand fluctuations in functional connectivity associated with mood. Second, we had a more limited sampling of participants with elevated mania ratings. This results from both the fact that these participants are more difficult to enroll as well as the limited ability to study these participants during the COVID-19 pandemic where significant restrictions were put on human subjects’ research and the ability of participants to leave the inpatient unit for assessment. Third, we did not quantify disease burden in terms of number of episodes, but instead asked for self-reported past-decade symptom burden (35); these measures may not be comparable and thus it is difficult to fully compare to studies that use the former. Fourth, medication is always a challenge in studies of psychiatric disorders with most patients taking multiple classes of medications. Most of the participants in this study were on two or more medications. We did explore the effects of medication in a univariate approach and did not find any overlap with the primary findings observed in this study, however, these analyses may be more vulnerable to confounding than our primary analyses with groups balanced by age, sex and subjective SES. Fifth, we conducted a whole brain exploration of functional connectivity of the vermis, which would provide a more comprehensive understanding of differences in vermal connectivity in bipolar disorder. However, this did reduce our power to detect significant findings only within the emotional control network. In addition, this study only explored functional connectivity of the vermis while structural connectivity was not assessed. Structural connectivity data would help to understand if these findings result from decreased apoptosis and increased structural connectivity between these regions or if the differences were solely functional. Finally, the size of some of the vermal regions of interest (V, Crus II, VIIb, and X) were relatively small given the acquired resolution of the resting state data. Multi-band acquisitions and higher field strength scanners open up the potential to acquired higher resolution (~2 mm isotropic) resting state functional connectivity data in the future.

In conclusion, the findings of this study support a significant role of the cerebellar vermis in bipolar disorder with abnormal functional connections between the vermis and cerebrum. The strength of some of these connections further differed by mood state. Additional work is needed to understand the nature of cerebellar connectivity in bipolar disorder and whether changing cerebellar connectivity should be leveraged for treatment. Nevertheless, given the location and depth of the vermis relative to the skull, the vermis is a potential target for modulation via transcranial magnetic stimulation. Indeed, there are ongoing studies using the vermis as a target for treatment of bipolar disorder, schizophrenia, and autism. While it is unclear if the same stimulation paradigm should be used as employed for the dorsal lateral prefrontal cortex (80), it is a potential novel therapeutic target that could help individuals with bipolar disorder to better maintain a euthymic mood state.

## Data availability statement

The datasets presented in this study can be found in online repositories. The name of the repository and accession number can be found below: https://nda.nih.gov/edit_collection.html?id=2810.

## Ethics statement

The studies involving human participants were reviewed and approved by the University of Iowa IRB01. The patients/participants provided their written informed consent to participate in this study.

## Author contributions

VM, JW, and JF contributed to the conception and design of the study. EB, GC, LS, JX, JS, and SS organized the database. AS, SJ, JS, and MV performed the image analysis. AS, GH, MV, EB, JL, and VM performed the statistical analysis. AS, GH, MV, JW, JF, and VM wrote the first draft of the manuscript. JR, AW, JF, and JW interviewed study participants and assessed symptoms. All authors contributed to the article and approved the submitted version.

## Funding

This study was supported by funding from the National Institute of Mental Health (R01MH111578) with studies conducted on equipment (S10OD025025 and S10RR028821) and facilities (UL1TR002537) supported by NIH. Some investigators received salary support from the Carver Foundation. JW was supported by NIH National Institute of Mental Health grant R01MH113325, NIH National Institute of Drug Abuse grant R01DA052953, the Roy J. Carver Charitable Trust, the Roy J. Carver Chair, a U.S. Department of Veterans Affairs Merit Review Award, and the U.S. Department of Veterans Affairs. This research was supported in part through High Performance Computing resources provided by the Information Technology Services team—Research Services at the University of Iowa, Iowa City, Iowa, directed by Joe Hetrick.

## Conflict of interest

The authors declare that the research was conducted in the absence of any commercial or financial relationships that could be construed as a potential conflict of interest.

## Publisher’s note

All claims expressed in this article are solely those of the authors and do not necessarily represent those of their affiliated organizations, or those of the publisher, the editors and the reviewers. Any product that may be evaluated in this article, or claim that may be made by its manufacturer, is not guaranteed or endorsed by the publisher.
