# Supplementary material for: Functional connectivity of the cerebellar vermis in bipolar disorder and associations with mood
Source: Front Psychiatry. 2023 May 5;14:1147540. doi: 10.3389/fpsyt.2023.1147540 (PMC10196126; doi:10.3389/fpsyt.2023.1147540)
Supplement: Supplementary file 1 [file Data_Sheet_1.PDF]

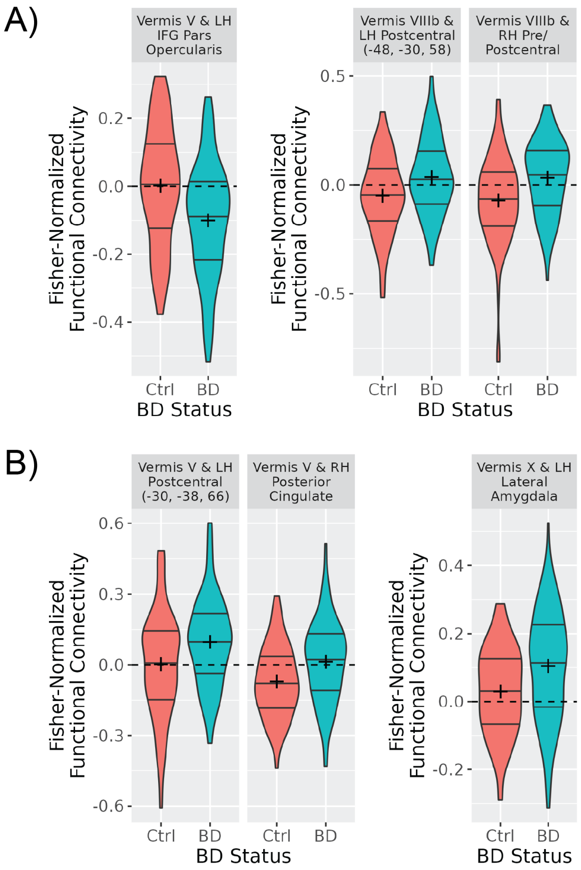

Supplemental Figure 1. Differences in cerebellar vermal connectivity in bipolar type I disorder as compared to controls identified using a linear regression analysis including covariates for age, sex, tSNR. This data is the same as shown in Fig 2, but is plotted as raw Fisher-Normalized Z-scores. **A)** Shows the functional connectivity for the bipolar and control groups for the significant connections shown in Fig 2A. **B)** Shows the functional connectivity for the bipolar and control groups for the trending connections shown in Fig 2C.

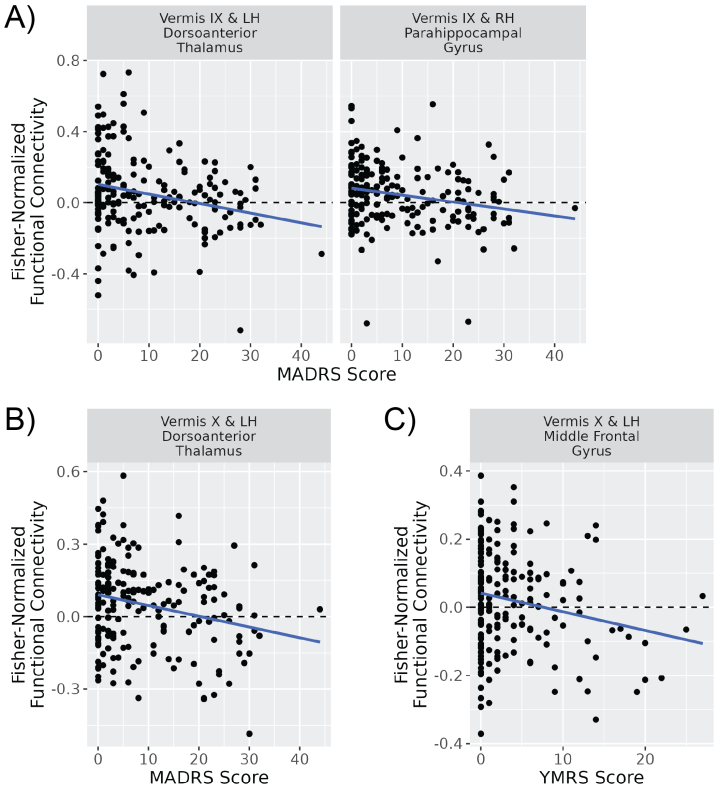

Supplemental Figure 2. Mood as assessed with the MADRS and YMRS scales is associated with vermal connectivity in bipolar type I disorder using a regression analysis including covariates for age, sex, and tSNR. The data are the same as shown in Fig 3 expect here they are plotted as raw Fisher normalized Z-scores. **A)** Shows the regression between MADRS and functional connectivity for the regions identified as significant in Fig 3A. **B)** Shows the regression between MADRS and functional connectivity for the regions identified as trending in Fig 3C. **C)** Shows the regression between YMRS and functional connectivity for the regions identified as significant in Fig 3E.

Supplemental Table 1. Number of statistical comparisons run for each vermal node. The total number of regions in the analysis was 445 and the number of comparisons was the number of remaining regions after removing those with a mean negative correlation in both the bipolar disorder and control cohorts.

Vermal Node	Number of Comparisons
I-IV	202
V	224
Crus II	235
VI	217
VIIb	203
VIIIa	257
VIIIb	242
IX	178
X	202

Supplemental Table 2. Regression analysis evaluating functional connectivity in participants with bipolar disorder on and off various classes of medications. Only regions identified with a p-value < 0.001 for the effect of diagnosis (Bipolar Disorder-Control) from the primary analysis were examined. Each connection and medication class were assessed separately. Covariates for age, sex, and tSNR were included. Medication class did not contribute significantly to the observed functional connectivity for these connections in the participants with bipolar disorder.

Seed Name and MNI Coordinate	Target Common Name MNI Coordinate	Medication Class	p-value	Beta Value
Vermis Lobule V (0, -48.5, -20)	Left Pars Opercularis (-52, 8, 14)	Antidepressants	0.225	-0.0458
		Antipsychotics	0.956	0.00183
		Sedatives	0.353	-0.0333
		Anticonvulsants	0.178	-0.0475
		Lithium	0.316	-0.0369
	Right Posterior Cingulate Gyrus (6, -52, 24)	Antidepressants	0.148	0.0562
		Antipsychotics	0.132	-0.0516
		Sedatives	0.812	0.00882
		Anticonvulsants	0.829	-0.00791
		Lithium	0.583	0.0209
	Left Postcentral Gyrus (-30, -38, 66)	Antidepressants	0.354	-0.0389
		Antipsychotics	0.930	0.00324
		Sedatives	0.446	0.0305
		Anticonvulsants	0.110	0.0625
		Lithium	0.516	-0.0267
Vermis Lobule VIIIB (0, -65, -45)	Right Pre/Postcentral Gyrus (60, -6, 26)	Antidepressants	0.150	0.0535
		Antipsychotics	0.271	-0.0361
		Sedatives	0.974	0.00117
		Anticonvulsants	0.561	-0.0203
		Lithium	0.758	0.0112
	Left Postcentral Gyrus (-48, -30, 58)	Antidepressants	0.902	-0.00479
		Antipsychotics	0.765	0.0102

		Sedatives	0.936	0.00297
		Anticonvulsants	0.648	0.0166
		Lithium	0.552	0.0225
Vermis Lobule X (0,-48,-35)	Left Lateral Amygdala (-26, -2,- 22)	Antidepressants	0.141	-0.0527
		Antipsychotics	0.301	0.0327
		Sedatives	0.497	0.0232
		Anticonvulsants	0.611	0.0171
		Lithium	0.591	-0.0188
